# Commentary on “Intra- and early postoperative predictors of delirium risk in cardiac surgery”

**DOI:** 10.1097/JS9.0000000000002740

**Published:** 2025-06-20

**Authors:** Shimeng Cui, Chao Wu, Xiaoxi Wang

**Affiliations:** a The First Affiliated Hospital of Dalian Medical University, Dalian, Liaoning, China; b Department of Clinical Laboratory Medicine, The First Affiliated Hospital of Dalian Medical University, Dalian, Liaoning, China.

*Dear Editor,*

We commend Itting *et al*^[1]^ for their rigorous prospective study on intraoperative and early postoperative predictors of postoperative delirium (POD) in cardiac surgery. The integration of multivariate regression and machine learning (ML) approaches, alongside a comprehensive analysis of modifiable perioperative factors, provides critical insights for developing targeted POD prevention strategies. While the study demonstrates strong scientific merit, we propose several methodological refinements to further enhance its robustness and clinical utility, particularly from a statistical perspective.

First, the manuscript highlights that multivariate logistic regression included factors significant in univariate analysis (e.g., age, ventilation time, surgical complexity), but the variable selection process—whether guided by clinical relevance, statistical significance (e.g., *P* < 0.05), or data-driven criteria (e.g., AIC/BIC)—remains unclear. Transparent reporting of model-building strategies is essential for reproducibility, especially when balancing statistical rigor with clinical interpretability^[2]^.For example, specifying how variables like “combined procedures” or “cardiac chamber opening” were prioritized based on prior evidence (e.g., their known association with cerebral microembolism) would strengthen the rationale for their inclusion. Additionally, acknowledging the use of AIC/BIC (as mentioned in the Results, Table 3) to optimize model parsimony could address potential concerns about overfitting, particularly given the complexity of intraoperative factors.

Second, while the authors presented well-organized multivariable analysis results, they did not report diagnostics for key assumptions of logistic regression. As Steyerberg *et al* have emphasized, omitting these diagnostic steps may compromise the robustness of results, particularly when interpreting log-transformed variables (such as ventilation time)^[3]^. We recommend including assessments of the logit linearity assumption and goodness-of-fit (e.g., Hosmer-Lemeshow test), which would enhance the methodological rigor and persuasiveness of the manuscript.

The ML-based decision tree and LASSO regression yield promising AUC values (0.7116 and 0.7407, respectively), but the manuscript could deepen its evaluation of model performance by including calibration plots or Brier scores to show how closely predicted POD probabilities align with observed outcomes, a key metric for clinical trust^[4]^. Assessing net benefit across clinically relevant risk thresholds (e.g., 10–30% POD risk) via decision curve analysis (DCA) would also quantify the models’ added value over simple heuristics, which is particularly important for guiding resource allocation in perioperative care (e.g., prioritizing early extubation for patients identified as high-risk by the LASSO model). By contextualizing ML results within clinical decision-making frameworks, the study would better inform the development of tools like ERACS protocols.

Itting *et al*’s work represents a significant step toward understanding POD pathogenesis in cardiac surgery, with actionable insights for reducing patient risk through optimized surgical and ventilatory protocols. By clarifying model-building strategies, validating regression assumptions, and enhancing ML model interpretability, the findings would achieve greater methodological rigor and clinical impact. We appreciate the authors’ contributions and hope these suggestions may further strengthen their important research.

## Data Availability

The data that support the findings of this study are all included in this article.

## References

[1] Itting PT Sadlonova M Santander MJ. Intra- and early postoperative predictors of delirium risk in cardiac surgery: results from the prospective observational FINDERI study. Int J Surg 2025;111:2872–85. 39903520 10.1097/JS9.0000000000002265 PMC12175825

[2] Zhang Z. Model building strategy for logistic regression: purposeful selection. Ann Transl Med 2016;4:111. 27127764 10.21037/atm.2016.02.15 PMC4828741

[3] Steyerberg EW Vickers AJ Cook NR. Assessing the performance of prediction models: a framework for traditional and novel measures. Epidemiology 2010;21:128–38. 20010215 10.1097/EDE.0b013e3181c30fb2 PMC3575184

[4] Van Calster B McLernon DJ van Smeden M Wynants L Steyerberg EW. Calibration: the Achilles heel of predictive analytics. BMC Med 2019;17:230. 31842878 10.1186/s12916-019-1466-7 PMC6912996

